# Percutaneous closure of native and residual ventricular septal defects using a diverse range of occluder devices: a single-centre experience

**DOI:** 10.1186/s43044-026-00741-8

**Published:** 2026-04-28

**Authors:** Ahmed R. S. A. Afifi, Suhair Shebani, Gregory Skinner, Atul Kalantre, Marinos Kantzis

**Affiliations:** 1 Pediatrics Department, Benha University, Benha, Egypt; 2 Congenital Heart Department, University Hospitals of Leicester NHS Trust, Leicester, UK

**Keywords:** Ventricular septal defect, Percutaneous closure, Congenital heart disease, Transcatheter closure, Interventional cardiology

## Abstract

**Background:**

Transcatheter closure of ventricular septal defects (VSDs) presents technical challenges in patients with complex anatomies and residual post-surgical defects. This study aimed to evaluate the feasibility, safety, and early outcomes of percutaneous VSD closure using a range of occlusion devices in a heterogeneous patient population.

**Methods:**

This was a retrospective single-centre observational study of patients who underwent percutaneous VSD closure between 2017 and 2024 at a tertiary congenital cardiac centre.

**Results:**

Twenty patients were included (12 females, 8 males) with a median age of 5.5 years (range 6 months–72.9 years) and median body weight of 18 kg (range 6.4–127 kg). Native VSDs were present in 14 patients and residual post-surgical defects in 6. Defect types included perimembranous (*n* = 13; including 3 Gerbode defects) and muscular (*n* = 7). Procedural success was achieved in 95% (19/20). Immediate complete occlusion occurred in 65% (13/20), while 7 patients had small residual shunts that remained haemodynamically insignificant at 1-year follow-up. Complications included one case of transient haemolysis and one case of ventricular ectopy requiring surgical device retrieval. No cases of complete heart block, device embolisation, or significant valve injury were observed during follow-up.

**Conclusion:**

Percutaneous VSD closure using a range of occlusion devices is feasible and safe across different anatomies and age groups, with high procedural success and low complication rates at one-year follow-up.

## Introduction

Ventricular septal defects (VSDs) are the most frequently encountered congenital heart lesions [1, 2]. While many small defects close spontaneously or remain clinically insignificant, a subset of patients requires intervention to prevent complications such as heart failure, pulmonary hypertension, or impaired growth. Surgical repair remains the standard treatment; however, transcatheter closure has become an increasingly accepted less invasive alternative in selected patients, including those with complex or higher-risk anatomies [3–7].

With advances in occluder technology and procedural techniques, the indications for transcatheter VSD closure have expanded beyond simple defects to include a broader range of anatomical substrates and patient groups [3–7]. Nevertheless, published data describing device performance across heterogeneous real-world scenarios—such as infants, adults, residual post-surgical defects, and uncommon morphologies including Gerbode-type defects—remain limited [8, 9].

This study reports a single-centre experience of percutaneous VSD closure across a diverse patient population, focusing on procedural characteristics, device usage (including cases where device choice was influenced by anatomical suitability and availability), and clinical outcomes. The aim is to contribute real-world data on feasibility and safety across a broad spectrum of VSD anatomies.

## Materials and methods

### Study aim

The study aimed to evaluate the feasibility, safety, and early outcomes of percutaneous VSD closure in a heterogeneous patient population and to report a single-centre real-world experience, including procedural characteristics and device usage across diverse anatomical settings.

### Study design

This was a retrospective, observational, single-centre cohort study conducted at the University Hospitals of Leicester, UK. All patients who underwent transcatheter VSD closure between 2017 and 2024 were included in the analysis.

### Study population

Patients of any age with native or residual post-surgical VSDs deemed haemodynamically significant were eligible for inclusion. All cases were discussed in multidisciplinary team meetings and listed for cardiac catheterization. Patients with post-myocardial infarction VSDs and those undergoing hybrid surgical-device closure were excluded to maintain a uniform transcatheter interventional approach.

Clinical, procedural, and follow-up data were collected from electronic medical records and catheterization reports.

### Procedure technique

All procedures were performed under general anaesthesia using combined fluoroscopic and transoesophageal echocardiographic (TOE) guidance. Vascular access was obtained via femoral artery and vein in most cases, with internal jugular venous access used selectively to improve alignment in muscular VSDs. Intravenous heparin (100 U/kg) was administered with activated clotting time monitoring.

**Fig. 1:**
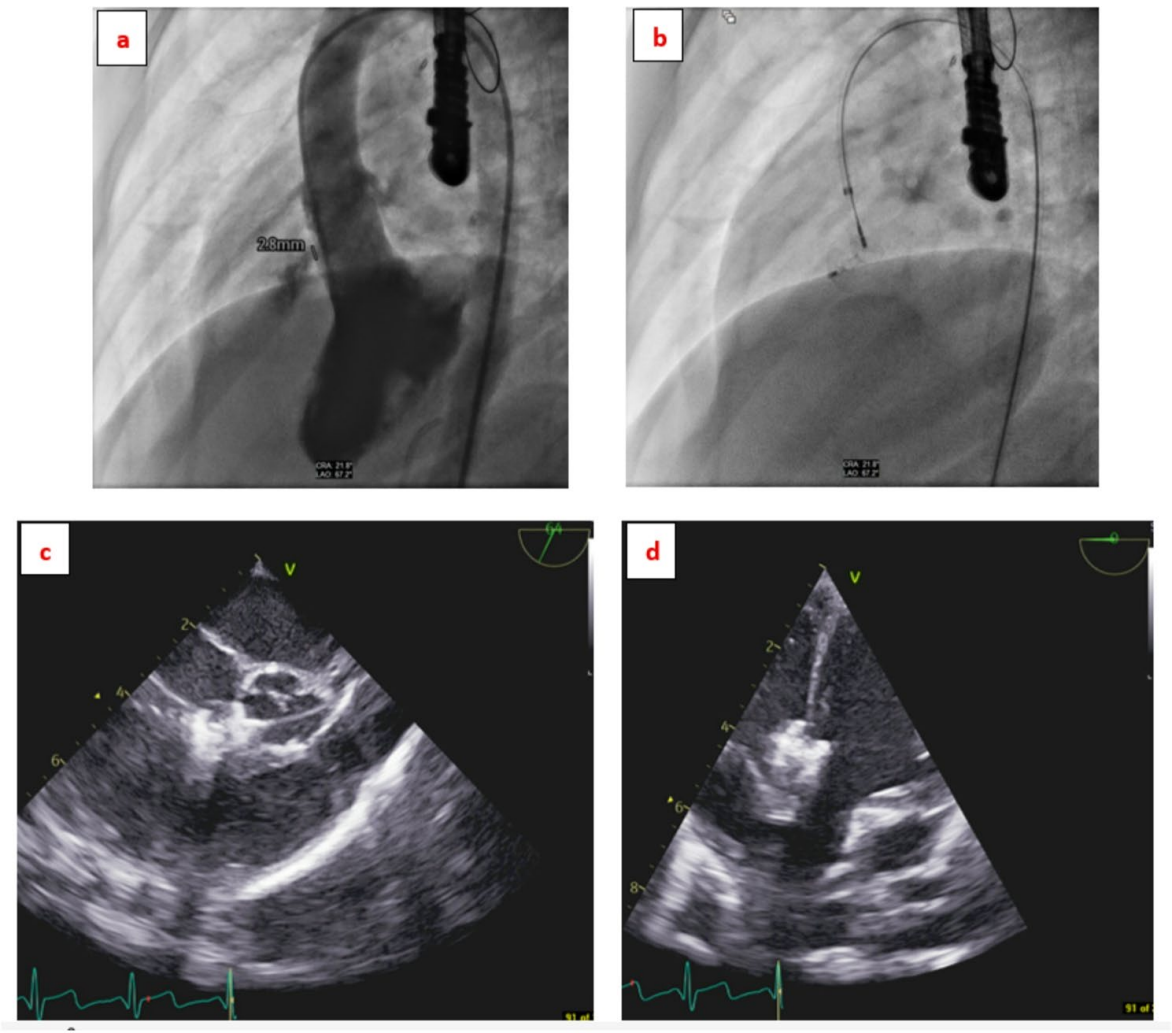
Perimembranous VSD around 3 mm diameter closed by MFO 6/4 device retrogradely. **a** LAO/ cranial angulation LV angiography showing VSD, **b** Fluoroscopy showing device in place attached to delivery cable via retrograde approach, **c** TOE showing device during deployment, **d** TOE showing the device in place after being released from the cable. *VSD* ventricular septal defect, *MFO* multi-functional occluder, *LAO* left anterior oblique, *LV* left ventricle, *TOE* trans-esophageal echo

**Fig. 2:**
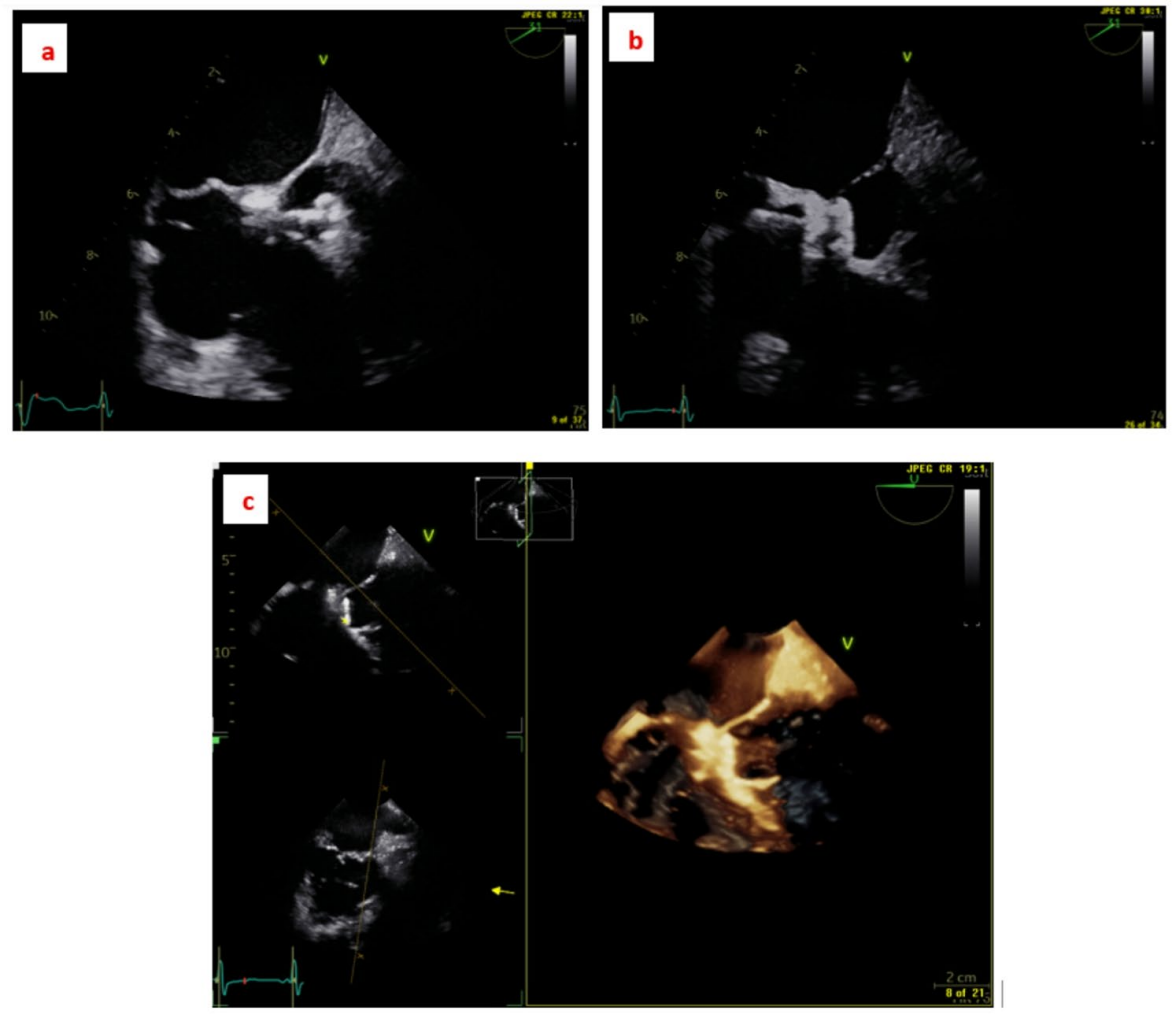
Gerbode type VSD 8 mm in diameter closed with 12/10 Multi-functional occluder device. **a** TOE showing the left ventricle disc deployed, **b** TOE showing both discs in good position before device release **c** 3D TOE showing the final position of the device post-release. *TOE* trans-esophageal echo

Left ventricular angiography and TOE were used to define VSD anatomy and guide device deployment. The defect was typically crossed from the left ventricle to the right ventricle, and an arteriovenous (AV) loop was established when required to facilitate delivery. In selected cases, including symmetric double-disc or multifunctional devices, a retrograde approach was used without loop formation (Fig. 1). Two cases with Gerbode-type defects were approached antegradely from the venous side without the need of creating an AV loop (Fig. 2).

Device selection was based on angiographic and echocardiographic measurements of the narrowest VSD diameter and defect morphology. Double-disc devices were generally selected equal to or slightly larger than the defect (0–2 mm), while single disc devices (duct occluders) were chosen with appropriate oversizing (at least 2 mm) to ensure stability and avoid interference with adjacent structures. Device deployment was performed under fluoroscopic and TOE guidance, with repeat angiography used to confirm position and residual shunt before release.

### Postoperative care, follow-up

All patients received aspirin (3–5 mg/kg/day, maximum 75–100 mg) for six months. Follow-up clinical and echocardiographic assessments were performed at 1, 3, 6, and 12 months to evaluate device position, residual shunt, ventricular function, and complications.

### Study outcomes

Primary outcomes were procedural feasibility and safety, defined by successful device implantation without major complications. Secondary outcomes included residual shunt, conduction abnormalities, device embolization, need for reintervention, and late clinical or echocardiographic findings at 1-year follow-up.

### Statistical analysis

Continuous variables are presented as median and range due to the small and heterogeneous sample size. Categorical variables are presented as frequencies and percentages. No inferential statistical tests were performed, as this was a descriptive observational study.

## Results

### Patients

Baseline characteristics are summarised in Table 1. The cohort included 20 patients with a median age of 5.5 years (range 6 months–72 years 11 months) and median weight of 18 kg (range 6.4–127 kg), reflecting a heterogeneous population. Native VSDs predominated, with a smaller proportion of post-surgical residual defects. Anatomical distribution included perimembranous and muscular defects, with a subset of LV–RA (Gerbode-type) lesions and multiple VSDs requiring dual-device closure. Closure was performed even for small defects, which were not always hemodynamically significant, when there were additional indications such as cusp prolapse, evidence of left ventricular volume overload on echocardiography, or post-surgical defects (e.g., Gerbode-type) with a history of arrhythmia.

**Table 1:** Baseline patient characteristics

Variable	Value
Total patients	20
Sex	Female 12 (60%), male 8 (40%)
Median age	5.5 years (range 6 months–72 years 11 months)
Median weight	18 kg (range 6.4–127 kg)
Patients with native VSD	14 (70%)
Patients with post-surgical VSD	6 (30%)
VSD type	Perimembranous 13 (65%) (*Gerbode type in 3 patients*) Muscular 7 (35%) (*Multiple VSDs in 4 patients*)
Median VSD diameter	6 mm (range 3–14 mm)

*VSD* ventricular septal defect

### Procedures

Procedural characteristics are summarised in Table 2. Vascular access and closure approach varied according to anatomy, with both antegrade and retrograde strategies used. A total of 24 devices were implanted across the cohort, and immediate complete occlusion was achieved in the majority of patients, while a minority had small residual shunts that were haemodynamically insignificant. A small number of lesions were not intervened upon due to limited clinical significance.

**Table 2:** Procedural characteristics

Variable	Value
Total procedures	20
Total VSDs treated	24
Patients with multiple VSDs	4 (20%)
Access site	Femoral 16 (80%), Internal jugular 4 (20%)
Approach	Antegrade 16 (80%), retrograde 4 (20%)
Devices implanted	Total 24 Amplatzer vascular plug II 10 Konar-MF 6 Amplatzer Duct Occlude II 5 Amplatzer Ductal Occluder I 1 Occlutech Ductal Occluder 1 Amplatzer Septal Occluder device 1
Fluoroscopy time (median, range)	Median 32.5 min, range 10–92 min

*VSD* ventricular septal defect

### Follow-up

Clinical and echocardiographic follow-up was performed at 1, 3, 6, and 12 months to assess device position, residual shunt, ventricular function, and complications.

### Outcomes

Outcomes are summarised in Table 3. Procedural success was high, with no cases of complete heart block, device embolisation, significant valvular regurgitation, or endocarditis during follow-up.

**Table 3:** Clinical outcomes and complications

Variable	Value	Details
Procedure success	19/20 (95%)	
Transient haemolysis	1 (5%)	Resolved by day 4 after Occlutech device
Surgical retrieval	1 (5%)	AVP II (22 mm), RVOT irritation, day 9 surgery
Complete heart block	0	
Device embolization	0	
Significant valve regurgitation	0	
Device malposition	0	
Endocarditis	0	
Immediate complete closure	13 (65%)	

*AVP II* Amplatzer vascular plug II

One patient (5%) developed transient haemolysis following closure of a 9 mm perimembranous VSD in the setting of heart failure. Initial attempts at closure using 10 mm and 14 mm Amplatzer Vascular Plug II (AVP II) devices were unsuccessful due to device instability and slippage within aneurysmal tissue. Definitive closure was achieved using a 15 × 12 mm Occlutech ductal occluder, which provided improved anchoring within the defect morphology. Post-procedurally, haemolysis resolved spontaneously by day 4 with conservative management.

**Fig. 3:**
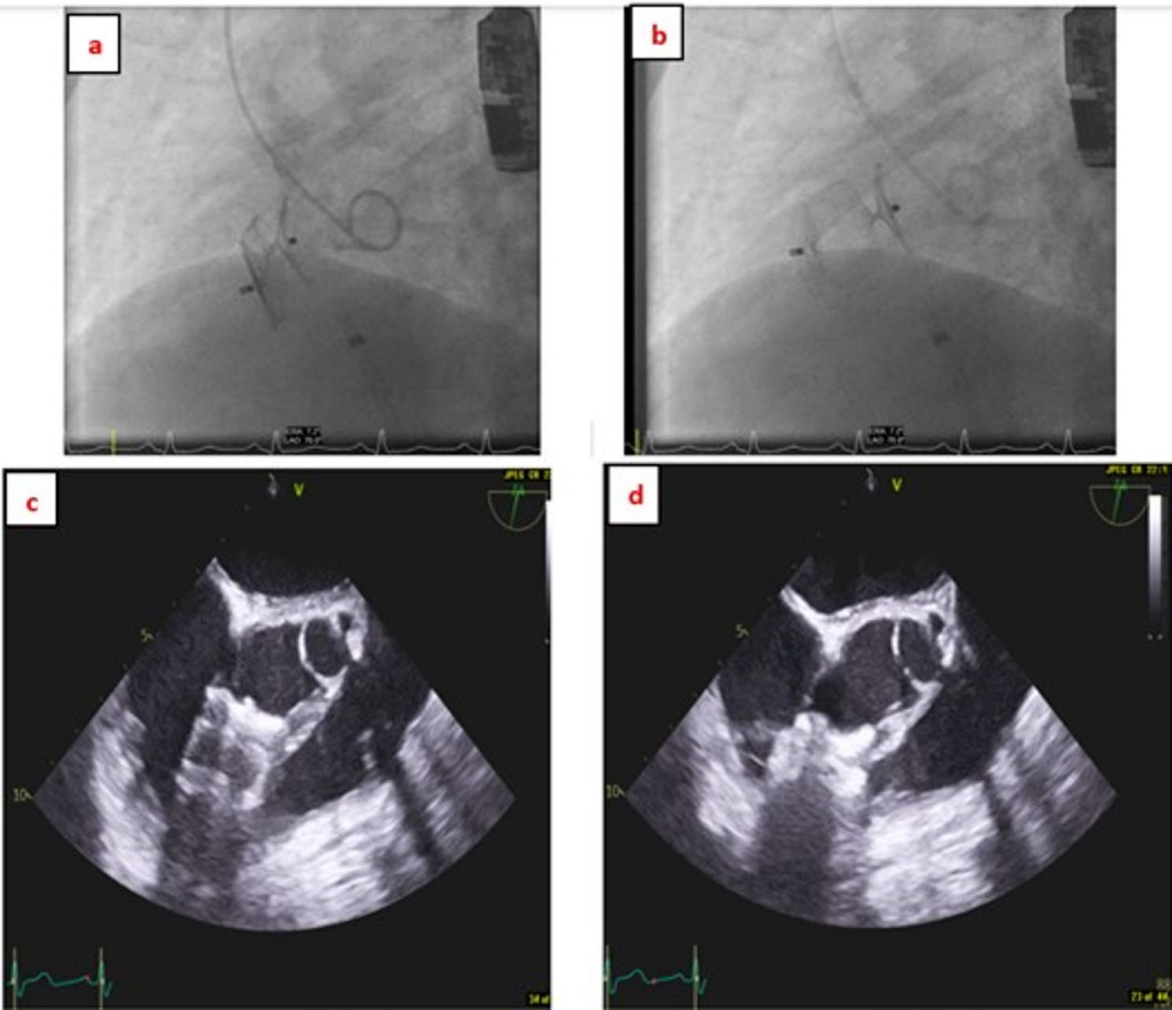
Perimembranous VSD (14 mm) closed using a 22 mm Amplatzer Vascular Plug II (AVP II). The device appeared elongated and demonstrated dynamic compression during systole, with protrusion toward the right ventricular outflow tract (RVOT), resulting in significant ventricular ectopy. **a**, **b** Fluoroscopic images showing cyclical shortening of the device during systole. **c**, **d** Corresponding transoesophageal echocardiography (TOE) images demonstrating the same dynamic changes. The device was surgically explanted after 9 days, and the VSD was closed successfully. This case highlights the importance of appropriate device sizing and caution when using longer devices in perimembranous VSDs with proximity to the RVOT

A second patient (5%) required surgical retrieval of a 22 mm AVP II device on day 9 after implantation in a 14 mm perimembranous VSD. Although immediate angiographic occlusion was achieved, echocardiography and fluoroscopy demonstrated significant device compression against the right ventricular free wall, resulting in mechanical irritation and persistent ventricular ectopic activity. This necessitated surgical device explantation and patch closure. The imaging findings are illustrated in Fig. 3. No residual haemodynamic complications were observed following surgery.

Detailed per-patient procedural, anatomical, and outcome data are provided in Table 4.

**Table 4:** VSD characteristics, device details, and outcome in patients who had device closure via cardiac catheterisation

Pt *n*	Primary diagnosis	VSD diameter, mm	VSD type	Device	Aortic rim from pm VSD, mm	Major complication	Minor complication	Closure side	Fluoroscopy time, minutes
1	ToF	8	Pm residual post surgery	ADO 14/12	8	None	None	Venous	35
2	Muscular VSD	5	Muscular	ADO II 6/4	–	None	Residual flow	Venous	72
3	Pm VSD	3	Pm Gerbode	ADO II 4/4	2.9	None	None	Venous	14
4	ToF	6	Pm Gerbode	ADO II 6/4	5	None	Residual flow	Venous	Unknown
5	PA, VSD	4	Muscular	ADO II 6/4	–	None	Residual flow	Venous	68
6	PA, VSD	3, 6	Muscular	ADO II 6/4, ASD 6	–	None	None	Venous	63
7	Pm VSD	9	Pm	Occlutech PDA occluder 15 × 12 (trials of AVPII 10 then 14 not successful, slipped)	6	None	Transient haemolysis	Venous	38
8	Pm VSD, dysplastic AV	10	Pm residual post surgery	AVP II 18 × 16	2.5	None	Residual flow	Venous	24
9	Pm VSD	6	Pm	AVP II 10	5	None	None	Venous	27
10	Pm VSD	3	Pm	MFO 7/5	3	None	Residual flow	Venous	30
11	Muscular VSD	7	Muscular	AVPII 8	–	None	Residual flow	Venous	46
12	Pm VSD	9	Pm	AVPII 14	2.5	None	None	Venous	17
13	Multiple VSDs	6, 8	muscular	AVPII 8, AVPII 10	–	None	None	Venous	92
14	Pm VSD	5.5	Pm	AVPII 12	7	None	Residual flow	Arterial	50
15	Pm VSD	14	Pm	AVPII 22	6	V ectopics, surgical retrieval	None	Venous	17
16	Multiple VSDs	3.7, 5	Muscular	AVPII 6, AVPII 10	–	None	None	Venous	84
17	Pm VSD	3	Pm	MFO 6/4	2.5	None	None	Arterial	15
18	Pm VSD	5.6	pm	MFO 8/6	4	None	None	Arterial	10
19	Multiple VSDs	8	Pm Gerbode	MFO 12/10	8	None	None	Venous	18
20	Interrupted arch, multiple VSDs	6, 3	muscular	MFO 10/8, MFO 6/4	–	None	None	arterial	68

*Pm VSD* perimembranous ventricular septal defect, *ADO* Amplatzer duct occlude, *AVP* Amplatzer vascular plug, *MFO* Multi-functional occluder, *PA* pulmonary atresia, *ASD* Atrial septal occluder

## Discussion

This study demonstrates the feasibility and safety of percutaneous VSD closure across a broad spectrum of patients, including infants, adults, residual post-surgical defects, and complex anatomies such as Gerbode-type defects, highlighting the expanding role of transcatheter approaches beyond traditional indications.

Known complications include heart block, valvular regurgitation, device embolization, and arrhythmias [3–7, 10–12]. In our series, careful device selection guided by anatomical characteristics and multimodality imaging was associated with a high procedural success rate and no permanent conduction abnormalities or device embolisation.

Device selection evolved over time. The Amplatzer Duct Occluder II (ADO II) was used in five cases (3–6 mm defects), including muscular and Gerbode-type VSDs, reflecting its suitability for small and delicate anatomies, with established high success and low complication rates reported in the literature [13–15].

From 2022 onward, AVP II use increased, particularly in tunnelled and aneurysmal defects due to its multi-lobed cylindrical design providing stable occlusion. However, limitations were observed in perimembranous positions, especially with larger devices. In one case, a 22 mm AVP II was intentionally oversized to achieve anchoring due to deficient aneurysmal tissue, resulting in RVOT protrusion and ventricular ectopy requiring surgical removal. In hindsight, an alternative device would likely have been more appropriate in this anatomy.

Published data support AVP II efficacy with high closure rates and low complication profiles [16], although most series used smaller devices with caution regarding size to avoid RVOT interaction. Other studies suggest careful sizing strategies up to 1–2 mm above the VSD size [17] and alternative device selection in cases with inadequate aneurysmal support [18], reinforcing the importance of careful anatomical assessment and device selection.

The Konar-MF occluder, introduced in 2023, provided a versatile option with a soft dual-disc design allowing deployment via either approach [19–21]. In our series, 6 devices were used in 5 patients (3–8 mm defects), with 4 cases closed retrogradely without AV loop formation, demonstrating stable deployment and no device-related complications.

Device choice was also influenced by anatomical complexity and availability. In a post–Tetralogy of Fallot repair with an 8–10 mm residual VSD, an ADO I was used after AV loop formation, achieving stable closure with minimal residual leak. In another case, after unsuccessful AVP II attempts (10 and 14 mm devices), a 15/12 Occlutech PDA device achieved successful closure without conduction or valvular complications under TOE guidance.

In a complex patient with pulmonary atresia, VSD, dextrocardia, and prior Rastelli repair, multiple residual defects were addressed. Following prior ADO II use, a large tunnel-like VSD was closed using a 6 mm Atrial septal occluder (ASD) device due to limited alternative options, with satisfactory positioning and outcome.

The ADO II additional sizes (ADO II AS) has recently emerged as a low-profile option for small perimembranous and post-surgical VSDs (2–4 mm), with encouraging early safety data [22]. Although not used in our cohort, it expands the available armamentarium for small defects.

Despite anatomical and technical heterogeneity, procedural success was high, with no device embolization or permanent conduction disturbances. Overall, our experience highlights that percutaneous VSD closure can be safely achieved using a wide range of devices selected according to anatomy and availability, with newer technologies further expanding treatment options for complex cases.

## Conclusion

Transcatheter VSD closure is a safe, feasible, and versatile strategy in both native and residual defects across all age groups. The heterogeneity in patient demographics, defect types, and device use in our cohort demonstrates the flexibility and real-world applicability of this approach. Broader adoption in selected cases is supported, with the caveat of thorough anatomical assessment and individualized planning.

## Limitations

This study is limited by its retrospective design, small sample size, and relatively short follow-up duration of one year. Larger, multicentre prospective studies with longer follow-up are required to further validate these findings.

## Data Availability

The datasets generated and/or analyzed during the current study are available from the corresponding author upon reasonable request.
